# Novel compound heterozygous *MYO7A* mutations in Moroccan families with autosomal recessive non-syndromic hearing loss

**DOI:** 10.1371/journal.pone.0176516

**Published:** 2017-05-04

**Authors:** Amina Bakhchane, Majida Charif, Amale Bousfiha, Redouane Boulouiz, Halima Nahili, Hassan Rouba, Hicham Charoute, Guy Lenaers, Abdelhamid Barakat

**Affiliations:** 1 Human Molecular Genetics Laboratory, Institut Pasteur du Maroc, Casablanca, Morocco; 2 Equipe MitoLab, INSERM U1083, CNRS 6015, Institut MitoVasc, Université d’Angers, Centre Hospitalier Universitaire d’Angers, Angers, France; Central South University, CHINA

## Abstract

The *MYO7A* gene encodes a protein belonging to the unconventional myosin super family. Mutations within *MYO7A* can lead to either non syndromic hearing loss or to the Usher syndrome type 1B (USH1B). Here, we report the results of genetic analyses performed on Moroccan families with autosomal recessive non syndromic hearing loss that identified two families with compound heterozygous *MYO7A* mutations. Five mutations (c.6025delG, c.6229T>A, c.3500T>A, c.5617C>T and c.4487C>A) were identified in these families, the latter presenting two differently affected branches. Multiple bioinformatics programs and molecular modelling predicted the pathogenic effect of these mutations. In conclusion, the absence of vestibular and retinal symptom in the affected patients suggests that these families have the isolated non-syndromic hearing loss DFNB2 (nonsyndromic autosomal recessive hearing loss) presentation, instead of USH1B.

## Introduction

Sensorineural hearing loss (SNHL) is the most prevalent human genetic sensory defect. It is estimated that globally 2 out of 1000 newborns have profound hearing loss [1]. Hereditary hearing loss is divided into two groups, syndromic and non-syndromic. To date, over 150 genes responsible for hearing loss have been identified, among which 70 are implicated in the non-syndromic hearing loss, whereas the others lead to syndromic presentations [2]. *MYO7A* is an unconventional myosin with a predicted 2215 amino acid sequence. Myosins are motor molecules that play an important role in intracellular movements; these proteins bind to actin filaments and use ATPase activity to generate the energy required for movements [3]. The human *MYO7A* gene contains 49 coding exons [4] and is expressed in the retina, lung, testis, kidney, and outer and inner hair cells of the cochlea [5]. In the latter, *MYO7A* is found in the actin-rich stereocilia bundles, cuticular plate, pericuticular necklace, and cell body [5]. *MYO7A* mutations are responsible for nonsyndromic autosomal recessive hearing loss (DFNB2) [6], autosomal dominant hearing loss (DFNA11) [7–9], and Usher syndrome [10]. Usher syndrome is an autosomal recessive disorder defined by the association of sensorineural hearing loss, retinitis pigmentosa (RP) and variable vestibular areflexia. Clinically, Usher syndrome can be classified into three types, USH type I (USH1), USH type II (USH2) and USH type III (USH3) [11]. Syndromic *MYO7A* mutations are inherited in a recessive manner, leading to a diagnosis of Usher type 1B (USH1B) [12,13], which is the most severe, and characterized by congenital profound hearing loss, prepuberal onset of retinitis pigmentosa, and vestibular dysfunction. However *MYO7A* variants have also been associated with USH2 [8,14], which is characterized by less severe features: moderate deafness without vestibular dysfunction and RP with postpubertal onset. Until now, only seven MYO7A mutations have been characterized in cases of recessive non-syndromic hearing loss: p.R244P in Chinese pedigree [15], a compound heterozygote mutation c.133-2A>G (IVS3nt-2A>G) and p.V1199insT in another Chinese pedigree [15], p.E1716del in Pakistani pedigree [16], p.R395H in Iranian pedigree [17], p.Cys652Glyfs*11 (p.C652fsX11) in Iraqi pedigree [18], p.P1887L in Palestinian pedigree [18].

In this study, we report the clinical, genetic and molecular characterization of two Moroccan families with Autosomal Recessive Non-Syndromic Hearing Loss (ARNSHL), thus disclosing the 7^th^ and 8^th^ DFNB2 families described to date. It is the first time that *MYO7A* mutations are identified with hearing loss in Morocco.

## Patients and methods

### Family enrolment and clinical evaluation

**Fig 1:**
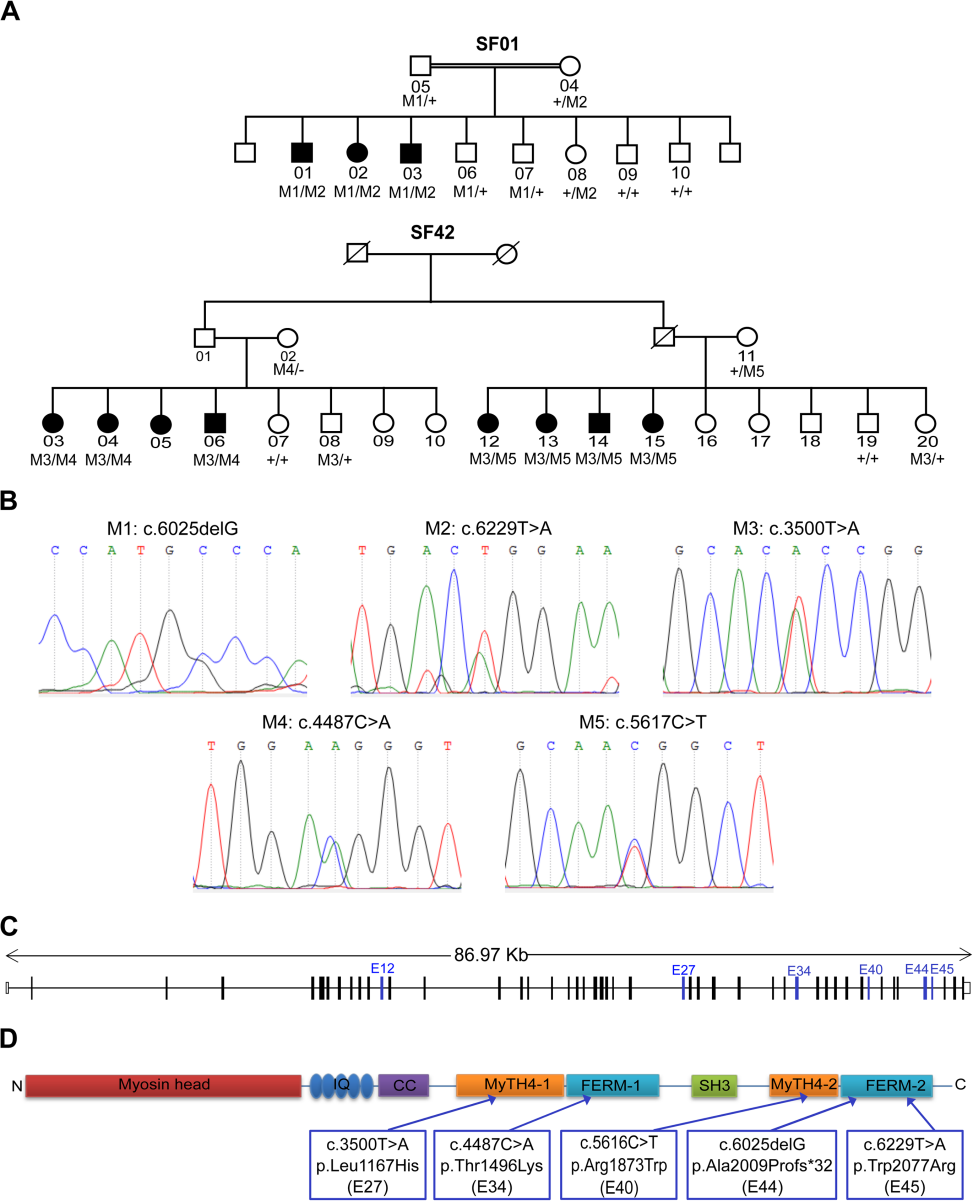
Molecular analysis of families SF01 and SF42 families. A: Pedigrees and genotype data for each member of the SF01 and SF42 families: M1 to M5: mutation1 to mutation 5. B: Electrophoregram presenting the *MYO7A* heterozygous mutations identified for each family: Family SF01 (mutations M1 and M2) and Family SF42 (mutations M3, M4 and M5). C: Mutated *MYO7A* exons. D: Mutated domains in the MYO7A protein.

Autosomal recessive non-syndromic sensorineural hearing loss has been ascertained in two large Moroccan families: SF01 and SF42 (Fig 1A). Written informed consent was obtained from all patients and controls, and the committee on research ethics of the Pasteur Institute of Morocco approved the genetic study. Audiological testing was assessed by pure tone audiometry, speech audiometry and tympanometry, using a pure tone audiometer and a tympanometer. Hearing impairment was classified as mild (20–40 dB), moderate (41–70 dB), severe (71–95 dB), and profound (>95 dB). Tandem gait and Romberg testing were performed for vestibular function evaluation.

In addition, 60 families with severe to profound congenital bilateral sensorineural hearing loss and 100 healthy controls from different regions of Morocco with no history of hearing loss, were included in this study to screen the identified *MYO7A* mutations.

### Whole exome sequencing

Genomic DNA was extracted from peripheral blood of the patient SF01.01 by phenol chloroform method following standard protocol [19]. Whole exome sequencing was performed at Otogenetics Corporation (Norcross, GA, USA) to identify the causative mutation in this patient. Genomic DNA was fragmented, end-repaired, and ligated with specific adaptors for library preparation using NEBNext reagents (New England Biolabs, Ipswich, MA, USA). Then, the resulting libraries were captured using Agilent Human exome V5 (51 Mb) capture kit and sequenced on a HiSeq 2000 platform (Illumina, San Diego, USA).

### Read mapping and variant analysis

Whole-exome sequencing generated about 25.2 million short reads, comprising 3.2 billion bases. After quality control, short reads were mapped to the human genome reference sequence (University of California Santa Cruz hg19) using the DNAnexus software package (DNAnexus, Inc, Mountain View, CA, USA). Short reads were mapped with an average exome coverage of 30x. Variant calling and annotation were performed using the DNAnexus software package. After quality control of the identified variants, the next step was to exclude all non-exonic and synonymous variants from further analysis. Then, the frequency of each variant was obtained from dbSNP database (version 132) and all variants with a frequency higher than 1% were filtered. Finally, the functional effects of missense variants were predicted using SIFT (Sorting Intolerant From Tolerant) and PolyPhen-2 (Polymorphism Phenotyping).

### Mutation confirmation and segregation analysis

To validate candidate mutations, segregation analysis was performed in all family members by Sanger sequencing, using the ABI prism Big Dye Terminator cycle sequencing Ready Reaction kit V 3.1 (ABI Prism/ Apllied Biosystems, Foster City, CA). The sequences were obtained through an ABI Prism 3100 Genetic Analyser (Applied Biosystem).

Additional unrelated control individuals were also sequenced, to determine the frequency of the candidate mutations. The primers used to screen the entire *MYO7A* coding region were designed using the Primer3 software (S1 Table).

### Molecular modelling

The 3D structure of the wild type MYO7A protein was modelled using CPHmodels-3.2, an automated homology modelling program [20]. Then, the FOLD-X program was used to generate mutated structures [21]. Structural analysis and visualization of the predicted structures were performed using YASARA software [22]. To analyse the impact of amino acid substitutions on MYO7A protein structure stability, we used MAESTROweb and SDM (Site Directed Mutator) bioinformatics tools [23,24].

## Results

### Clinical evaluation

Audiological testing was completed to document the degree of hearing loss, which disclosed severe to profound congenital bilateral hearing loss in all the affected members from the SF01 family. However, in the SF42 family, audiological evaluation showed mild progressive hearing loss in the left branch, while the deafness was congenital and severe in the right one. Normal vestibular function was revealed by caloric tests performed on patients SF01.02, SF42.03 and SF42.14. The visual acuity was normal in both eyes in all affected persons, and fundus examination in patient SF01.02 showed intact fundi, as late as 36 years of age (Table 1).

**Table 1:** Clinical characteristics of patients SF01.02, SF42.03 and SF42.14.

Patient	Age	Sex	Onset of hearing loss	Hearing threshold (dB)	Fundus examination	Visual acuity
SF01.02	36	F	Congenital	70	Normal retina, blood vessels and optic disc	20/20
SF42.03	33	F	Congenital	60	ND	20/20
SF42.14	30	M	Congenital	90	ND	20/20

### Whole exome sequencing

Whole-exome sequencing (WES) of a DNA sample from patient SF1.01 generated a total of 122725 single nucleotide variants and 8622 short insertions and deletions (indels). To select candidate causative mutations, we focused on non-synonymous variants, frameshift in/del and variants in splice sites. Common variants with a frequency greater than 1% were filtered based on dbSNP137, 1000 Genomes Project and HapMap project databases. Considering an autosomal recessive mode of inheritance, homozygous or compound heterozygous variants were selected for further analysis. The remaining variants were filtered using Polyphen-2 and SIFT bioinformatics tools; only variants predicted as damaging were retained (S2 Table). Finally, we performed variant screening in candidate genes by prioritizing previously reported hereditary hearing loss genes.

WES analysis revealed two novel compound heterozygous mutations in *MYO7A* coding region. The c.6025delG deletion in exon 44 leads to a frameshift and a premature stop codon, truncating the last 206 amino-acids of the protein (p.Ala2009Profs*32). The c.6229T>A missense substitution occurs in exon 45, resulting in an amino acid substitution (p.Trp2077Arg). Sanger sequencing confirmed the co-segregation of these *MYO7A* mutations with the pathological phenotype, as they were present in all affected individuals at compound heterozygous state. The parents were heterozygous carriers of the c.6025delG (mother) and the c.6229T>A (father) mutations, whereas unaffected family members had only one of these mutations at heterozygous state, or none (Fig 1A and 1B).

In addition, sequencing of all *MYO7A* exons in 60 families with hereditary hearing loss, led to the identification of further mutations in SF42 family. All affected persons presented the c.3500T>A mutation (p.Leu1167His) in addition to one of the following mutations: c.5617C>T (p.Arg1873Trp) on the right side of the family and c.4487C>A (p.Thr1496Lys) on the left side of the family, at compound heterozygous state (Fig 1A and 1B). These *MYO7A* mutations were absent in 100 healthy controls.

Among the four missense mutations, three were predicted to be deleterious (p.Leu1167His, p.Arg1873Trp and p.Trp2077Arg) as revealed by multiple bioinformatics software, including SIFT [25], Polyphen [26], CONDEL [27], MutationAssessor [28] and MutationTaster [29]. In contrast, SIFT and Mutation Assessor predicted that the p.Thr1496Lys missense mutation is not damaging, while other prediction tools classified this substitution as disease causing (Table 2).

**Table 2:** Characteristics of *MYO7A* mutations.

cDNA Change	Protein Change	dbSNP rs ID	MAF in Exome Variant Server and Exac	SIFT	Polyphen 2	Condel	Mutation Assessor	Mutation Taster	DDIG-in
M3: c.3500T>A	p.Leu1167His	-	-	Deleterious	Probably damaging	Deleterious	High	Disease causing	-
M4: c.4487C>A	p.Thr1496Lys	rs373651847	0.0079 8.328e-06	Tolerated	Possibly damaging	Deleterious	Low	Disease causing	-
M5: c.5617C>T	p.Arg1873Trp	rs397516321	- 8.817e-06	Deleterious	Probably damaging	Deleterious	Medium	Disease causing	-
M1: c.6025delG	p.Ala2009Profs*32	-	-	-	-	-	-	Disease causing	Disease causing
M2: c.6229T>A	p.Trp2077Arg	-	-	Deleterious	Probably damaging	Deleterious	Medium	Disease causing	-

**Fig 2:**
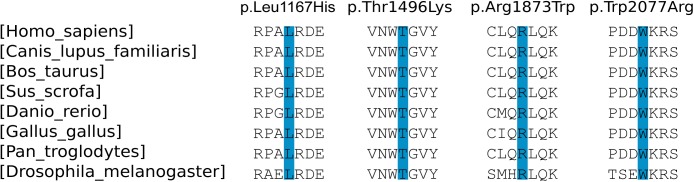
Alignment of *MYO7A* amino acid sequences from different species. The mutated amino-acids which are highly conserved residues are highlighted in blue.

All missense mutations as well as the frameshift mutation p.Ala2009Profs*32 are located in the MyTH4 domain and the FERM domain (Fig 1C and 1D). In addition, multiple sequence alignment of MYO7A orthologous proteins from different metazoan species showed that the missense mutations affected highly conserved residues (Fig 2).

### Structure modelling

**Fig 3:**
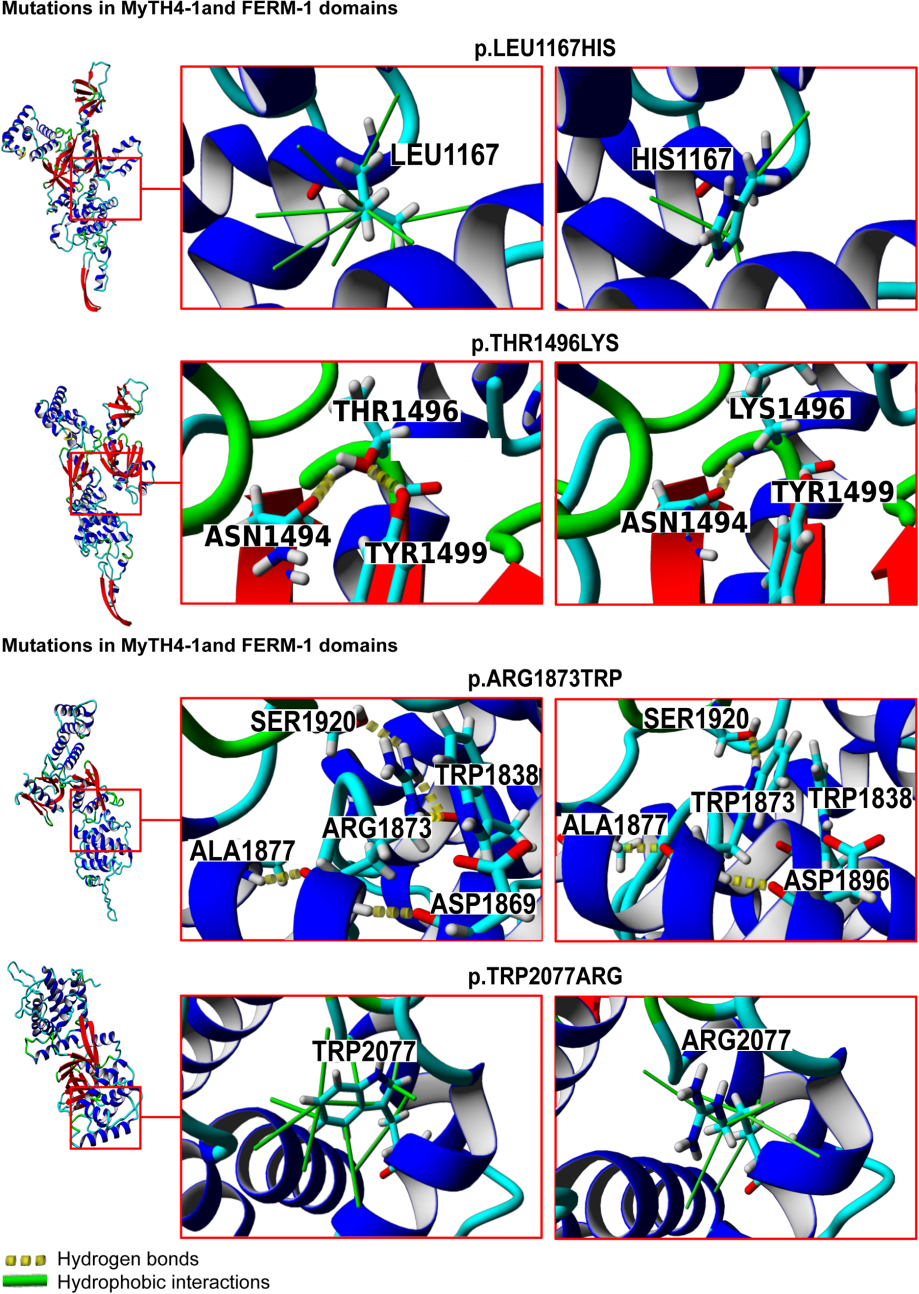
Structural and functional impacts of *MYO7A* missense mutations, as predicted by molecular modelling and amino acid conservation analysis. Hydrogen bonds and hydrophobic interactions predicted by the Yasara software. Yellow dotted lines represent hydrogen bonds, and green lines represent hydrophobic interactions.

A 3D structure of MYO7A head domain was constructed based on the crystal structure of the myosin head domain and of the heavy chain 2 protein (MYH2) (PDB ID: 2XEL). The sequence identity between the target and template proteins is 43.07%, and the constructed model covered 98% of the target sequence. The crystal structure of MYO7A MyTH4-FERM (PDB ID: 3PVL) was used as a template to build a molecular model of the second MyTH4-FERM tandem domain. The identity and convergence between the target and template proteins were 26.54% and 87%, respectively (Fig 3).

The substitution of p.Leu1167His is likely to disrupt the hydrophobic interaction between Leu1167 and its neighbouring residues. The p.Thr1496Lys substitution may induce a loss of hydrogen bonds, thus altering the interaction between residues Thr1496 and Tyr1499. The mutation p.Arg1873Trp is predicted to remove the hydrogen bond between Arg1873 and Trp1838 residue. The amino acid change p.Trp2077Arg may disrupt the hydrophobic interactions between Trp2077 and the neighbouring residues.

The bioinformatics tools SDM and MAESTROweb have been used to predict MYO7A missense mutation effects on the protein stability. Two mutations, p.Leu1167His and p.Trp2077Arg, were predicted to be destabilizing by both tools. The p.Arg1873Trp mutation was found to be highly stabilizing causing protein malfunction. The p.Thr1496Lys is predicted to be destabilizing by SDM, in contrast MAESTROweb predicted that this substitution may stabilise the protein (Table 3).

**Table 3:** Mutation effects on MYO7A 3D structure stability.

cDNA Change	Protein Change	SDM	MAESTROweb
c.3500T>A	p.Leu1167His	Destabilising	Destabilising
c.4487C>A	p.Thr1496Lys	Destabilizing	Stabilising
c.5617C>T	p.Arg1873Trp	Highly stabilising	Stabilising
c.6229T>A	p.Trp2077Arg	Highly destabilising	Destabilising

## Discussion

In the present study, we performed whole-exome sequencing on a Moroccan family with autosomal recessive, non-syndromic hearing loss (ARNSHL), which did not show mutation in the most common genes involved in the Moroccan deaf population, like *GJB2* [30], *LRTOMT2* [31], *TBC1D24* [32] and *TMC1* [33]. We identified two novel *MYO7A* mutations that prompted us to explore *MYO7A* sequence in additional Moroccan families. In a second family including two branches, we further revealed three novel *MYO7A* mutations. All five *MYO7A* mutations that are predicted to be pathogenic, co-segregated with the phenotype and were absent in 100 healthy individuals. The *MYO7A* gene encodes a protein belonging to the unconventional myosin superfamily. The MYO7A protein is characterized by the presence of a head domain in the N-terminal region, this domain contains an ATP and an actin binding sites, followed by the neck domain, containing five isoleucine-glutamine (IQ) motifs, which function as binding sites for partners. The tail domain has an important role in the regulation of MYO7A movement, it contains two myosin tail homology 4 (MyTH4) domains, two band 4.1-ezrinradixin-moesin (FERM) domains and an SH3 domain [34]. *MYO7A* has been reported to be involved in multiple sensory functions, such as vision and hearing. Consequently, mutations in this gene are responsible for the Usher syndrome type 1B, and the homozygous c.1687G>A mutation predicted to result in aberrant splicing was described in a large Moroccan consanguineous USH1 family [35].

In this study, two novel mutations at compound heterozygous state c.6025delG (p.Ala2009Profs*32) and c.6229T>A (p.Trp2077Arg) were identified in patients from family SF01. The c.6025delG deletion leads to a frameshift mutation predicted to introduce a premature stop codon truncating a large part from the second FERM domain. Within the same family, the c.6229T>A, resulting in a substitution of tryptophan, a neutral hydrophobic amino acid residue, by arginine, a positive charged hydrophilic amino acid at 2077 position, may lead to a change in the protein structure by disrupting hydrophobic interactions between the alpha helix containing Trp2077 and the neighbouring residues. Interestingly, in the family SF42, we identified three different compound heterozygous mutations in the two branches of the family. All affected members share the c.3500T>A (p.Leu1167His) mutation at compound heterozygote state, and one of the following mutations: c.5617C>T and c.4487C>A. These missense mutations are located in conserved regions and are putatively damaging, while not found in 100 Moroccan controls. Since the p.Thr1496Lys amino acid change found in the left branch of the family is predicted to be mild, whereas the p.Arg1873Trp amino acid change is predicted to be severe (Table 1), different auditory phenotypes were observed in the two branches: mild and progressive in the left branch, while being congenital severe in the right one. In the first MyTH4 domain, the substitution of Leucine, a hydrophobic uncharged residue, to Histidine a positively charged residue at position 1167 is likely to disrupt the hydrophobic interactions between Leu1167 and its neighbouring residues. The missense mutation p.Thr1496Lys affects the first FERM domain by replacing a neutral residue with a positive charged amino acid. This substitution may eliminate a hydrogen bond between Thr1496 and Tyr1499, thus perturbing the interactions between the adjacent beta strand containing residues Asn1494 and Tyr1499. The substitution p.Arg1873Trp involves the change of a positive charged hydrophilic amino acid to a neutral hydrophobic amino acid. It occurs within the second MyTH4 domain and may change the protein folding by perturbing the hydrogen bond between Arg1873 and Trp1838 residues. The possible pathogenic effect of the mutations identified in this study on the MYO7A protein structure and multiple bioinformatics programs, amino acid conservation analysis and molecular modelling reinforce function. All mutations were located in functionally important protein domains, and are suspected to disrupt the normal MYO7A function, preventing its interaction with other proteins, such as Harmonin (USH1C) and Cadherin 23 (CDH23), SANS (USH1G) [36,37]. The MYO7A and SANS proteins form a complex involved in the formation of the stereo-cilia of hair cells, while the MyTH4-FERM tandem domains mediate the interaction between both proteins [36]. Through its first and second FERM domains; MYO7A binds to the integrin b5 subunit, and regulates cell adhesion and migration in non-stere-ocilia cells [38].

In conclusion, we describe here the first two Moroccan families with *MYO7A* compound heterozygote mutations causing hearing loss. In addition, our genotype-phenotype correlations suggest that these mutations are leading to the DFNB2 rather than USHIB clinical presentation. Elucidation of the molecular causes of these recessive disorders is crucial to implement prevention programs, especially for highly consanguineous populations.

## Supporting information

S1 Table List of primers used to amplify and sequence the MYO7A coding regions. (DOCX)

S2 Table List of the remaining causative variants from the exome sequencing data of the patient SF01.01 before prioritization of reported hereditary hearing loss genes. (XLSX)
