# Supplementary material for: Novel compound heterozygous MYO7A mutations in Moroccan families with autosomal recessive non-syndromic hearing loss
Source: PLoS One. 2017 May 4;12(5):e0176516. doi: 10.1371/journal.pone.0176516 (PMC5417485; doi:10.1371/journal.pone.0176516)
Supplement: S1 Table — (DOCX) [file pone.0176516.s001.docx]

| **Gène** | **Amorces** | **% GC** |
| --- | --- | --- |
| MYO7A_2F/R | F :5’-AGCCAGGCTCAAGGCTTC-3’  R :5’-CCTAGGCAGGAATTTTCCAAG-3’ | 59 ,42 |
| MYO7A_3F/R | F :5’- GCCTCTGCTAAGTCATCCATAAAA-3’  R :5’- GCTCCCCCTGCACTCAAACC-3’ | 57.60 |
| MYO7A_4F/R | F :5’GAGCGGTCCTTGAGGGGTAGAG-3’  R :5’- GGCGGGGTGGGGCGAGGTG-3’ | 68 |
| MYO7A_5F/R | F :5’- CTAAGGAGGCCCGATTCTGG-3’  R :5’-AAGGGGAGGTGATTTTCTACATTG - 3’ | 56 |
| MYO7A_6/7F/R | F :5’- AGATGGGGGAGCTGGTGGAT-3’  R :5’-CGGTGGGTTTCATGGTGGGATTTC - 3’ | 60.75 |
| MYO7A_8F/R | F :5’- CCCTCTGTGCTGGAAATC-3’  R :5’- GCAAGAAGCTGGCACCTGAG- 3’ | 57.40 |
| MYO7A_9F/R | F :5’- GGCACCAGCACCCCCAGATG-3’  R :5’- TCAGCACAGCGACACCACACC- 3’ | 61.46 |
| MYO7A_10F/R | F :5’- TGACCTGGGGAAGCATTTAG-3’  R :5’- CTCAACCCTTCAGAGGGAC- 3’ | 60.13 |
| MYO7A_11F/R | F :5’-GCGGGGCTGTCAAGGAGAG -3’  R :5’-GGAAGGCCCCAGAGGAGCAT - 3’ | 66.34 |
| MYO7A_12F/R | F :5’-TTTCACACGGCACTTTGTTC -3’  R :5’-GAAGGAAAGGGAGAGGGTCC - 3’ | 55.93 |
| MYO7A_13F/R | F :5’-CTTGCTGGGCCTCCGTGTCC -3’  R :5’-CTATACTTGAATTGGCCCCTGAGC - 3’ | 56.69 |
| MYO7A_14F/R | F :5’- AGAAGAGACAGGGGGCAAAGACAT-3’  R :5’- AGAAAGCCCTGGAAATAGATAGCA- 3’ | 50,66 |
| MYO7A_15F/R | F :5’-GATGGCCCCTCACTTTCTCTA -3’  R :5’-GCACCCCAGCCATATCCCCAGTTA - 3’ | 57,93 |
| MYO7A_16F/R | F :5’-AAACTTCAAATACCGCCCTG -3’  R :5’-CCATTCCCCAAAGGGAC - 3’ | 62.53 |
| MYO7A_17F/R | F :5’-AGATCCCGGTGCCTGTCC -3’  R :5’-AGCCCCGCCCATGGTCCTGATA - 3’ | 62 |
| MYO7A_18F/R | F :5’-GTTTTGCAATCATACCATCCA -3’  R :5’-CTCCTCCAGCCACACCACA - 3’ | 60.90 |
| MYO7A_19F/R | F :5’-GGCCCAACTGAGTTCTTGAC -3’  R :5’-TGTGCACTTTCATAGGTG - 3’ | 55  44.4 |
| MYO7A_20/21F/R | F :5’-AGAAGTTATGTGCCTTGCCC -3’  R :5’- GGTCTGGTCCCAGGTATGTG- 3’ | 60.21 |
| MYO7A_22F/R | F :5’-AAAGTCATGCCCAGTTCC -3’  R :5’-GCCATTCTGAAGGATGGTAG - 3’ | 50  50 |
| MYO7A_23F/R | F :5’-TCCAGAGGTGGGGAAGTCAG -3’  R :5’-CCCGGACCCAGTTCATCTC - 3’ | 63,49 |
| MYO7A_24F/R | F :5’-GATGGGGTCGTACCCTGTTG -3’  R :5’- GGGCACCGCAGTGAAGAC- 3’ | 61,56 |
| MYO7A_25F/R | F :5’-CCATGCGGGAGGGGGTGTCT -3’  R :5’-GGCAGGCAGGGGTCGGTGAG - 3’ | 60,91 |
| MYO7A_26F/R | F :5’- GGACACCCTGTAAGCTTCAC-3’  R :5’-ACAGTGTCCACTCCTGCTCC - 3’ | 61,25 |
| MYO7A_27F/R | F :5’- TGACAGTGATGGGGAGCC-3’  R :5’- AACTAGCTAGCAGCGAAGCC- 3’ | 60,20 |
| MYO7A_28F/R | F :5’- GACCGGGGCTGTTCCTGTG-3’  R :5’-GGCCTGCCCCAAGACGAG - 3’ | 63,85 |
| MYO7A_29F/R | F :5’- CCCGACTGGCTGGTGCTGTGA-3’  R :5’- ATCCTTTGGGGGCTTTTCTGTTCC- 3’ | 61,72 |
| MYO7A_30F/R | F :5’-GGACAAGCAGTGTCCCAGTG -3’  R :5’- CTCCCAAAGTGCTGGGATTAC- 3’ | 60  52.4 |
| MYO7A_31F/R | F :5’- GCTGGGCCTCCGTTTTCTGTCTGA-3’  R :5’- CTCCCGCCATCTCGCCTCTCC- 3’ | 62.61 |
| MYO7A_32F/R | F :5’- TGAGAGCTACAGGAGGCAGG-3’  R :5’-AATGAAGGAAGGGCCACTG - 3’ | 62.16 |
| MYO7A_33F/R | F :5’- TGCAGACAGATGGGAGCAG-3’  R :5’-GGCTGGAGCTACAGAGCAAG - 3’ | 55,20 |
| MYO7A_34F/R | F :5’- CACGTAGCGAGTTTGTGCTC-3’  R :5’-CCAATCACGTGCAACCAG - 3’ | 57.57 |
| MYO7A_35F/R | F :5’-GGAATTAGACAGGCAAAGGAGAAG -3’  R :5’-TGCAGAACTGTGAGCCAAATAAAC - 3’ | 57.85 |
| MYO7A_36F/R | F :5’- CCCAAGGGCAATGAGGAG-3’  R :5’-TGAGCACTGCCCCAACTTTA - 3’ | 55.81 |
| MYO7A_37F/R | F :5’- TCAGAGACCCCAAGGAGGAACG-3’  R :5’-TGGGGTGGGTGCAGTGACATCT - 3’ | 59.96 |
| MYO7A_38F/R | F :5’- GCAGGGCAGGGAAGAGCA-3’  R :5’-CCCAGCACAAGGCACCAAATC - 3’ | 65.65 |
| MYO7A_39F/R | F :5’- CATGCCCATTTTGTGAGTGTGC-3’  R :5’- TGGGGCCAAGCTAGGATGTGA- 3’ | 61.89 |
| MYO7A_40F/R | F :5’-GAGGGACGGTGCTGCTGTGATGAG -3’  R :5’-GGAGAGGCCCAGCAAAGTGAAGAA - 3’ | 65.73 |
| MYO7A_41F/R | F :5’-CTCCGAGGGCACTTGTGTTCTTCA -3’  R :5’- ACGAGGCGGCAAATGTCAGGTG- 3’ | 59.95 |
| MYO7A_42F/R | F :5’-TTTTGTTAAATGCCATGCCC -3’  R :5’-GATGCCAGTGCCCTCTTG - 3’ | 59.89 |
| MYO7A_43/44F/R | F :5’-GAGTCTAAGGCAGCGTTCAGTGTC -3’  R :5’-TGGCAAGGTCAGTGGGTCTCT - 3’ | 58.53 |
| MYO7A_45F/R | F :5’-GCGTTGGGGGTCTTGGTGTGGTG -3’  R :5’-GGGTGGGCGGCCTCATCAAGT - 3’ | 63.72 |
| MYO7A_46F/R | F :5’- AGACGGGGCTGGAGTGG-3’  R :5’-GCTGGGACAAGGCTGAGG - 3’ | 61.55 |
| MYO7A_47F/R | F :5’-GTGCAGGGTCCAGGAGGCTTTTTA -3’  R :5’-AGGGCTTGTGGGGTTCGTTCTG - 3’ | 56.91 |
| MYO7A_48F/R | F :5’-ACCCAACAGCCTCCTAGACTTCCT -3’  R :5’-CATGACCCTTGCCCACGAC - 3’ | 62.46 |
| MYO7A_49F/R | F :5’-TTCAATTGTGCAGATGGGAGATAA -3’  R :5’- CAGAGGGGTGGATGGAGGGAAGT-3’ | 57.89 |
